# Design of a 2D Melting Curve–Based Multiplex PCR Assay for Detection of SARS‐CoV‐2/RSV/Influenza A‐B

**DOI:** 10.1155/cjid/6687705

**Published:** 2025-12-05

**Authors:** Murat Sayan, Ayse Arikan, Tahaberk Atesoğlu

**Affiliations:** ^1^ PCR Unit, Research and Education Hospital, Kocaeli University, Kocaeli, Turkey, kocaeli.edu.tr; ^2^ DESAM Research Institute, Near East University, Nicosia, TRNC Mersin 10, Turkey, neu.edu.tr; ^3^ Department of Medical Microbiology and Clinical Microbiology, Near East University, Nicosia, TRNC Mersin 10, Turkey, neu.edu.tr; ^4^ Department of Medical Microbiology and Clinical Microbiology, Kyrenia University, Kyrenia, TRNC Mersin 10, Turkey; ^5^ Department of Molecular Biology and Genetics, Faculty of Arts and Sciences, Yıldız Technical University, Istanbul, Turkey, yildiz.edu.tr; ^6^ Diagnotech Biotechnology and Life Sciences Research, Istanbul, Turkey

**Keywords:** diagnosis, Influenza A-B, molecular beacon, multiplex PCR, RSV, SARS-CoV-2

## Abstract

**Background:**

Recent advances in detecting respiratory pathogens have allowed for the simultaneous identification of multiple agents, enabling quick and accurate diagnosis to start timely treatment. This study aimed to design a novel two‐dimensional (2D) multiplex reverse transcription quantitative PCR (RT‐qPCR) assay. This assay allows for the concurrent detection of SARS‐CoV‐2, RSV, and Influenza A‐B using molecular beacon technology in a single‐well format.

**Methods:**

We used 550 nasopharyngeal swab samples from the Kocaeli University, Research and Educational Hospital, PCR laboratory, along with synthetic plasmids for SARS‐CoV‐2, RSV, Influenza A‐B, and internal control (RNase P). DNA products generated after amplification interacted with intermediate probes containing specific enzymatic cleavage sites and fluorescent markers, producing characteristic melting temperature (Tm) values for melting curve analysis.

**Results:**

Distinct Tm values were identified for SARS‐CoV‐2 (72°C), RSV (66°C), Influenza A (56°C), Influenza B (68°C), and internal control (80.5°C). The accuracy was confirmed by testing laboratory‐confirmed samples and synthetic plasmids, with no cross‐reactivity or false positives observed.

**Conclusions:**

This melting curve–based assay can differentiate among various pathogens within a single well and fluorescence channel by utilizing the unique Tm of each target. Consequently, this novel assay may serve as a cost‐effective, high‐throughput PCR testing method compared to traditional diagnostics.

## 1. Introduction

Trends in acute respiratory infections have been increasing globally since January 2025, particularly in Asia, Europe, Central America, and West and Central Africa [1]. These increases were reported to be caused by common respiratory viruses, including seasonal influenza viruses, respiratory syncytial virus (RSV), Human Metapneumovirus (hMPV), and *Mycoplasma pneumoniae* [1]. As of January 20–26, 2025, influenza, RSV, and SARS‐CoV‐2 positivity rates were detected at 49%, 6%, and 2%, respectively, in Europe [2]. In New York City, approximately 15,000 and 3000 cases of influenza and RSV, respectively, were detected as positive by the end of the first week of January 2025 [3].

Recent advances in detecting respiratory pathogens have focused on detecting multiple pathogens simultaneously, using techniques such as nucleic acid amplification tests, immunoassays, and biosensors [4]. Simultaneous detection of multiple pathogens enables accurate identification of the agent in a short time and the timely initiation of treatment, thereby preventing disease progression and reducing mortality [5]. The Coronavirus Disease 2019 (COVID‐19) pandemic has underscored the urgent need for rapid and decentralized diagnostic solutions, particularly during global health crises when supply chains may be disrupted. Although real‐time polymerase chain reaction (PCR) is still the most powerful tool and used as gold standard, especially for detecting viral pathogens in clinical samples due to its high specificity and accuracy, in recent years, there is an increased need for more advanced and sophisticated designs to detect multiple pathogens that are circulating widely in the community and pose a risk [6, 7]. Melting curve–based quantitative polymerase chain reaction (qPCR) design is a new technology that enables the simultaneous identification of up to 72 different pathogens, based on the principle of the melting curve generated by real‐time monitoring of changes in fluorescence signals as double‐stranded DNA (dsDNA) melts during the heating process [8–10].

Melt curve analysis is based on the denaturation properties of dsDNA products formed after PCR, monitored during a controlled temperature increase. The DNA double helix dissolves at a certain temperature (Tm—melting temperature) at which 50% of the base pairs are separated. This melting temperature is a unique value for each PCR product, depending on the base content, length, and structural properties of the DNA sequence [11]. Real‐time PCR devices monitor the melting of fluorescently labeled dsDNA as the temperature is gradually increased after amplification. Changes in the resulting fluorescent signal appear as peaks on the −dF/dT (derivative of fluorescence change with temperature) graph. Specific to each target, these melt peaks enable the separation of multiple targets with high resolution in a single channel. Additionally, melt curve analysis provides quality control benefits, including verification of amplification specificity, detection of primer dimers, and early detection of potential PCR inhibitors. The presence of internal control (IC) targets in the same well ensures test validity and increases the reliability of clinical results. This technical approach is also defined in the literature as high‐resolution melt curve (HRM) analysis, and the process is simple, applicable, rapid, and cost‐effective in identifying multiple pathogens and genotypes [8, 12].

In this study, we aimed to design and develop a novel two‐dimensional (2D) multiplex reverse transcription quantitative polymerase chain reaction (RT‐qPCR) assay that enables simultaneous detection of SARS‐CoV‐2, RSV, and Influenza A‐B (Inf A‐B) using molecular beacon technology, in a single‐well format.

## 2. Materials and Methods

For the design, 550 archived laboratory‐confirmed nasopharyngeal swab samples stored at −80°C were used. The samples were obtained from the PCR laboratory at Kocaeli University Research and Training Hospital. Additionally, synthetic plasmids for SARS‐CoV‐2, RSV, Inf A‐B, and an IC (RNase P) were utilized. Samples that tested positive with the DIAGNOTECH RESPLEX Influenza A, Influenza B, RSV, SARS‐CoV‐2 real‐time PCR kit (CE‐IVD‐approved commercial test) were used for the design. The limit of detection (LoD) values of the Diagnotech reference kit are given: 23.08 copies/reaction for SARS‐CoV‐2, 26.02 copies/reaction for Influenza A, 45.65 copies/reaction for Influenza B, 24.87 copies/reaction for RSV A, and 25.96 copies/reaction for RSV B. The samples were designed to contain target viral genome sequences and were obtained commercially as plasmids. The synthetic plasmids were obtained commercially from Eurofins Genomics (Ebersberg, Germany).

### 2.1. Primer and Mediator Probe Design

In this study, we used our designed Taq sequence and the added restriction enzyme cutting sites. Primers and mediator probes were designed to amplify and detect the viral RNA target with high specificity and efficiency for SARS‐CoV‐2, RSV, Inf A‐B, and IC (Table 1). Target gene regions were identified through a literature search and retrieved from NCBI GenBank (FASTA) format. Sequence specificity was confirmed using NCBI BLAST; mfold, Tm, and hairpin analyses were performed for molecular beacon structures. For RSV, the primers in this article were used: https://pcrbio.com/app/uploads/App-Note-Test-panel-for-SARS-CoV-2-winter-viruses-using-multiplex-RT-qPCR-v1.1.pdf. The primers and target genes used are as follows, respectively: Influenza A–F; 5′‐ ACCGAGGTCGAAACGT‐3′ R; 5′‐GAG​AGC​CTC​AAG​ATC​TGT​GT ‐ 3′ primers and M ‐ M ‐ segment 7; Influenza B‐ F; 5′‐ TTTGGAGACACAATTGCC ‐ 3′ R; 5′‐ GCTGAGTCTAGGTCAAAT ‐ 3′ and M ‐ M‐segment 7; RSV ‐ F; 5’ ‐ GCA​AAT​ATG​GAA​ACA​TAC​GTG​AAC​A ‐ 3′ R; 5′‐ GCACCCATATTGTWAGTGATGCA ‐ 3′ and Matrix protein; SARS‐CoV‐2 ‐ F; 5′‐ TTA​CAA​ACA​TTG​GCC​GCA​AA ‐ 3′ R; 5′‐ GCGCGACATTCCGAAGAA ‐ 3′ and N_2_; IC ‐ F; 5′‐ AGATTTGGACCTGCGAGC ‐ 3′ R; 5′‐ GAG​CGG​CTG​TCT​CCA​CAA​GT ‐ 3′ and Human RNase P.

**Table 1:** Primer nucleotide sets and detected viral RNA targets for SARS‐CoV‐2, RSV, Inf A‐B, and IC in the study.

Pathogen	Primer set	Target gene
Influenza A	F: 5′‐ACCGAGGTCGAAACGT‐3′ R: 5′‐GAGAGCCTCAAGATCTGTGT‐3′	M‐Segment 7
Influenza B	F: 5′‐ TTTGGAGACACAATTGCC‐3′ R: 5′‐ GCTGAGTCTAGGTCAAAT‐3′	M‐Segment 7
RSV^∗^	F: 5′‐ GCAAATATGGAAACATACGTGAACA‐3′ R: 5′‐ GCACCCATATTGTWAGTGATGCA‐3′	Matrix protein
SARS‐CoV‐2	F: 5′‐ TTACAAACATTGGCCGCAAA‐3′ R: 5′‐ GCGCGACATTCCGAAGAA‐3′	N2
IC	F: 5′‐ AGATTTGGACCTGCGAGC‐3′ R: 5′‐ GAGCGGCTGTCTCCACAAGT‐3′	Human RNase P

Abbreviations: IC: internal control, F: forward, R: reverse, M: matrix, and N: nucleocapsid.

^∗^Primers were used from https://pcrbio.com/app/uploads/App-Note-Test-panel-for-SARS-CoV-2-winter-viruses-using-multiplex-RT-qPCR-v1.1.pdf.

A Taq sequence was placed at the 5′ ends of the primers to correspond to a specific region of the mediator probe. The HiScript III U + One Step qRT‐PCR probe kit, a commercial kit manufactured by Vazyme Biotech (Nanjing, China), was used in the study for high‐throughput reverse transcription and real‐time PCR. A restriction enzyme (*PspGI*) (NEB, Catalog No: R0611S; 100% active in CutSmart buffer, 2–3 times more active at 85°C than at 75°C, half‐life of 2 h at 95°C), cutting sequence (CCTGG) containing a phosphorothioate modification was added to the region following this sequence. After amplification, the DNA opposite the Taq sequence was cut, and the released Taq fragment was bound to the complementary stem primer sequence, forming a peak in the melt curve analysis with a specific Tm. The design allowed the *Psp*GI enzyme to bind only to the complementary sequence, but the phosphorothioate modification in the modified chain prevented complete separation of the substrate.

In the study, commercially designed mediator probes (Bio‐Rad CFX96, Hercules, CA, USA) were used to analyze amplified sequences. Mediator probes were designed to carry a fluorescent molecule (e.g., HEX, FAM, ROX, or Cy5) at one end and a silencing molecule (e.g., BHQ1 or BHQ2) at the other end. Complementary sequences were added to both ends of the probes to enable them to anneal to themselves and detect fluorescent signal changes during sequence‐specific binding. The process began with the binding of the mediator probe to the primer‐derived amplification product. The mediator probe sizes were as follows: SARS‐CoV‐2, 30 bp; Inf A‐B, 16 bp; RSV, 24 bp; and IC, 36 bp. A specific region of the amplification product was cut by the restriction enzyme (PspGI), and this fragment hybridized with the probe sequence. During hybridization, the silencing between the two ends of the probe (quencher effect) was eliminated, and the fluorescent molecule produced a signal with laser excitation (Bio‐Rad CFX96, Hercules, CA, USA). The resulting fluorescent signal was analyzed in detail using the melt array method.

### 2.2. Reverse‐Transcription and qPCR Analysis

Reverse‐transcription and qPCR were performed in a single‐reaction protocol using Vazyme One‐Step RT‐qPCR Mix (Vazyme Biotech, Nanjing, China). The PspGI enzyme, with high‐temperature stability (up to 95°C), was used at a concentration of 0.25 units/μL for each reaction. The concentrations of primer and mediator probes were optimized as 0.4 μM and 0.2 μM, respectively. All reactions were carried out following the thermal cycling protocol as follows.

Reverse‐transcription: 50°C, 5 min, initial denaturation: 95°C, 2 min, PCR cycle (40 cycles, 95°C, 5 s, 55°C, 10 s, 72°C, 30 s, 72°C, 5 min, 54°C, 1 min), and melt curve analysis: temperature scanning between 55°C and 95°C.

### 2.3. Results Analysis

After qPCR, melt curve analyses were performed. Specific melting temperatures for each pathogen enabled the identification of the target viral RNA (see Table 2). As the design was melt‐array‐based intra‐ and interassay variations were assessed using Tm (melt curve) values rather than Ct values. Each sample was tested in three replicates across three different runs.

**Table 2:** Specific melting temperatures for SARS‐CoV‐2, RSV, Influenza A‐B, and IC.

Target	Dye	Tm,°C
Influenza A‐B	FAM or HEX	56 (FAM) or 68 (HEX)
RSV	FAM	66
SARS‐CoV‐2	FAM	72
IC	ROX or HEX	68 (ROX) or 80.5 (HEX)

Abbreviations: Tm: melting temperature; RSV: respiratory syncytial virus; IC: internal control.

### 2.4. Ethical Approval

This study was conducted with the permission of the Kocaeli University Noninterventional Clinical Research Ethics Committee, with the decision number KU GOKAEK‐2023/18.11 and Project No: 2023/351.

## 3. Results

In the study, a total of positive SARS‐CoV‐2 samples (*n* = 224, 41%), Influenza A (*n* = 83, 15%), Influenza B (*n* = 17, 3%), RSV (*n* = 57, 10%), negative samples (*n* = 162, 29%), and coinfections (RSV + SARS‐CoV‐2) (*n* = 7, 2%) were involved. With the design, increased specificity and reliable results in melt curve analysis were achieved after amplification against SARS‐CoV‐2, RSV, and Inf A‐B. Melt peak analyses were performed for pathogens, yielding specific melting peaks. No signal was observed in negative control (no‐template control, NTC) reactions, which were run to identify false negatives. Additionally, no peaks were observed in the mixed PCR reactions run with rhinovirus, *Streptococcus pyogenes,* and *M. pneumoniae*. Therefore, no false positivity was detected. These samples were from patients previously identified as positive for these pathogens and stored in our archives.

The applicability of this technology, especially for molecular diagnosis of multiple viral infections, enables faster diagnosis and the development of effective treatment strategies. This is especially acceptable compared to conventional PCR, because of the many features listed in Table 3.

**Table 3:** Comparison between the newly designed system and commercial PCR.

Characteristic	Commercial assay The DIAGNOTECH™ RESPLEX Influenza A, Influenza B, RSV, SARS‐CoV‐2 real‐time PCR kit (a CE‐IVD‐approved commercial test)	Designed assay
Number of wells	8	1
Pipetting	Preparing the master mix and adding it to wells	Adding the ready master mix in a single well
Internal control	In one well	In each well
Consumable cost	High	Low
Number of targets	1/Channel	6/Channel
Time	Both preparation and time per patient are ∼ 2 h	Both preparation and time per patient are less than 2 h

Figure 1 presents the melting peaks obtained for SARS‐CoV‐2, RSV, and Inf A‐B. Specifically, characteristic melting temperatures were observed for SARS‐CoV‐2 (Tm: 72°C), Influenza A and SARS‐CoV‐2 (Tm: 56°C and 72°C); Influenza A, RSV, and SARS‐CoV‐2 (Tm: 56°C, 66°C, and 72°C); Influenza A, IC and SARS‐CoV‐2 (Tm: 56°C, 80.5 °C, and 72°C); IC and SARS‐CoV‐2 (Tm: 68°C, and 72°C); Influenza A (Tm: 56°C); Influenza B, RSV, IC and SARS‐CoV‐2 (Tm: 68°C, 66°C, 80.5°C and 72°C).

Figure 1Melt curve analysis of amplified targets. (a) SARS‐CoV‐2 peak; (b) INF A and SARS‐CoV‐2 peaks; (c) INF A, RSV and SARS‐CoV‐2; (d) INF A and SARS‐CoV‐2 (FAM), IC (HEX); (e) IC (ROX) and SARS‐CoV‐2 (FAM); (f) INF A (FAM); (g) RSV and N2 (FAM), IC, and INF A (HEX). The graph was generated using Bio‐Rad CFX Maestro Software (Version 2.3) with raw data obtained from the Bio‐Rad CFX96 real‐time PCR instrument. The default software parameters were applied for melt curve analysis. The graph was directly exported from the software without any additional processing or editing.

**Figure:**
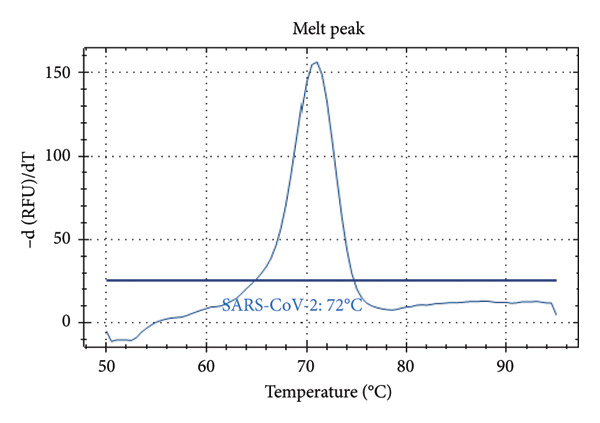
(a)

**Figure:**
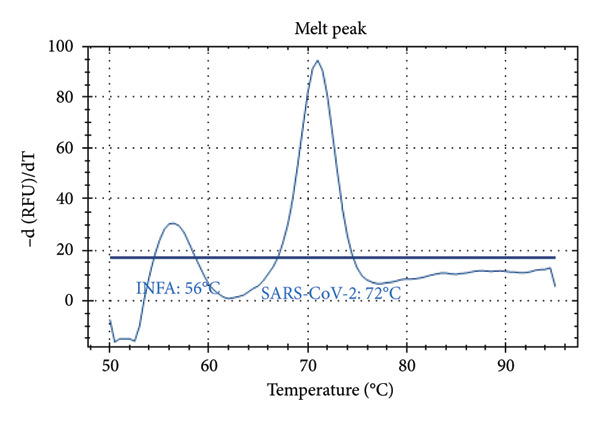
(b)

**Figure:**
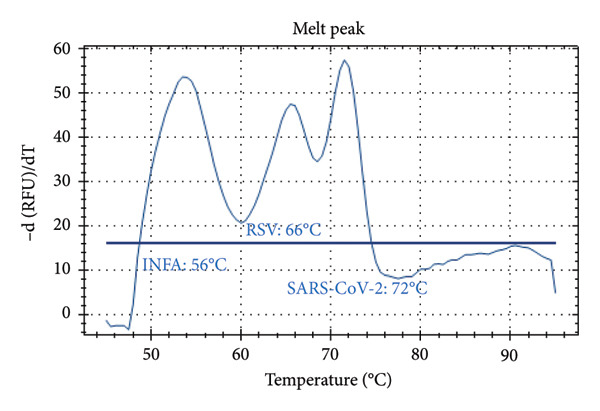
(c)

**Figure:**
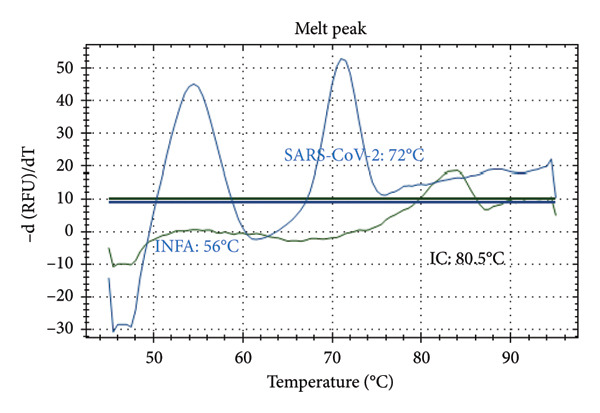
(d)

**Figure:**
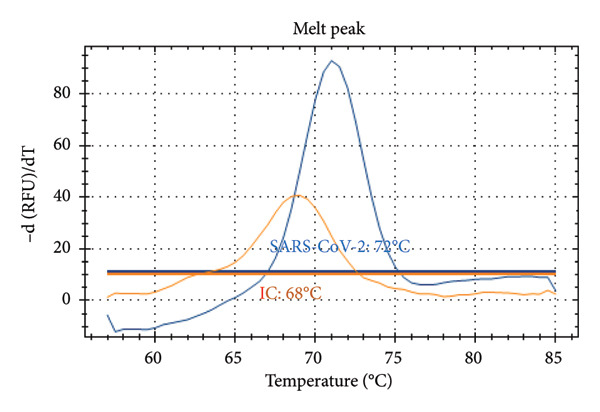
(e)

**Figure:**
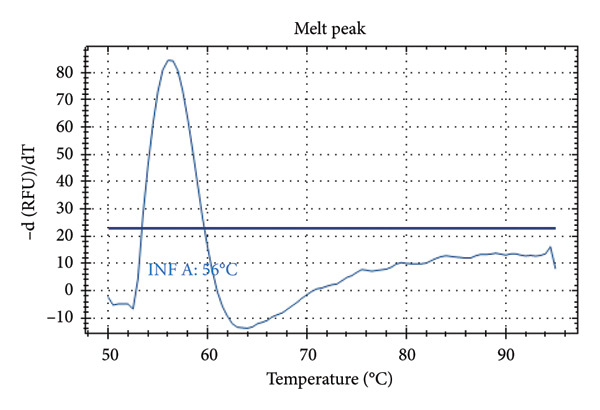
(f)

**Figure:**
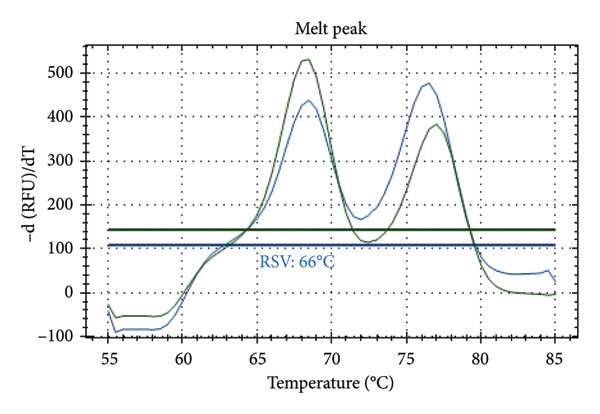
(g)

## 4. Discussion

Detecting multiple pathogens simultaneously with high accuracy in molecular diagnosis undoubtedly accelerates the clinical decision‐making process. It reduces costs, especially for respiratory tract infections, sexually transmitted diseases, and gastrointestinal syndromes. Melt curve‐based multiplex qPCR systems, developed for this purpose, allow the identification of multiple targets in a single fluorescence channel by utilizing the specific Tm of each target. Designing targets according to Tm values at approximately 6°C intervals (e.g., 55, 61, 67, 73, 79, 85°C) prevents potential cross‐signal interference and provides reliable identification with a temperature resolution of ±0.1°C. By design, we predicted that a Tm of approximately 6°C would provide clear and robust peak separation between targets. While there is no universal standard requiring a °C difference, literature and practical experience indicate that smaller differences, such as ≈1°C, are generally sufficient to distinguish melting peaks (e.g., in multiple HRM for *Escherichia coli* ST131, Tm between 0.99°C and 1.69°C provided acceptable and clear separation) [12]. Additionally, practical design guidance using Melt recommends targeting a minimum of 1.5°C–2°C between melt peaks in SYBR Green multiplex analyses. Another study reported three distinct peaks in a melting curve‐based real‐time PCR assay, highlighting their clear separation. The authors state, “The melt curve plot generated by the assay showed three distinct and well‐separated peaks,” suggesting Tm values were sufficient [13]. This statement supports that a ∼6 °C difference in a system provides robust separation and demonstrates that ideal ranges can be used for clear signal separation in typical HRM systems. Therefore, our use of a Tm of ∼6°C increases specificity and minimizes the risk of peak overlap. We could have used a Tm of ∼4°C; however, to avoid interference between Tm peaks and enhance the separation’s sharpness, we set it to 6°C in the study. This wide Tm range enables simultaneous detection of six targets using a single fluorescence channel, significantly increasing multiplex capacity. With this design, six targets can be detected in a single fluorescence channel, while 24 targets can be detected in a single well in a 4‐channel device. A 96‐well PCR device can produce results for 94 patients and two controls in ∼2 h. Most conventional kits operate with a single target in a single channel; some detect multiple targets in a single well. Our system reduces the number of pipetting steps and standardizes the application of ICs. ICs can be integrated into various channels, as demonstrated in the study.

Moreover, Tm changes were observed in the melt curve analysis with an average range of 0.2°C–1.0°C. In conventional multiplex qPCR systems, the user prepares separate mixtures for each well, performs pipetting, and adds an IC. This approach increases workload and user errors. Since the IC is not added to all wells, erroneous results may be missed. On the other hand, in this newly developed technique, a ready‐made master mix with fixed content is provided to the user. The user only transfers this mix to the wells and adds the samples. There is no need to use a separate mix for each well. With this new approach, user errors are minimized, ICs are 100% effective on every sample, and consumable use and time are significantly reduced. For instance, in a PCR panel with 8 wells, 10 patients are tested at a time, requiring the device to be run multiple times for a total of 94 samples. However, it is possible to run 94 patients and two controls using consumables that work with only 10 patients and two controls on the new system.

In the melt curve analysis, temperature shifts are desired in the range of 0.2°C–0.5°C. However, in multiplex melt curve analyses, shifts of several degrees in melting temperatures can be observed due to amplification of different targets in the same reaction [14, 15]. These Tm shifts are caused by primer‐probe interactions, changes in amplification efficiency, and factors related to the sample matrix. They are minimized through proper Tm‐range design and high‐resolution analysis algorithms [16]. In this study, melting peaks for SARS‐CoV‐2, RSV, and Influenza A and B were accurately designed and play a critical role in detecting pathogens. In addition to these pathogens, the new design can directly be adaptable to the identification of many infectious diseases such as; (i) HPV genotyping: Differentiation of high/low risk types such as types 6, 11, 16, 18, 31, 33, 35 etc., (ii) Respiratory panel: SARS‐CoV‐2, Inf A‐B, RSV, adenovirus, rhinovirus, metapneumovirus etc., (iii) Sexually transmitted infections: *Chlamydia trachomatis*, *Neisseria gonorrhoeae, Mycoplasma genitalium, Trichomonas vaginalis* etc., (iv) Gastrointestinal panel: *Salmonella*, *Shigella*, Enterrohaemorrhagic *Escherichia coli, Campylobacter*, *C. difficile*, norovirus etc., (v) Meningitis panel: Bacterial, fungal and viral meningitis samples, and (vi) many other syndromic panels.

### 4.1. Limitations

Our study has some limitations. First, the assay was not designed to detect Influenza A and B separately. The identification of A and B influenza viruses, which exhibit similar clinical symptoms, enables the exclusion of bacterial infections and facilitates the prompt decision‐making for appropriate treatment and isolation. It prevents unnecessary antibiotic use and guides antiviral treatment decisions. This suggests that identification without distinguishing between Influenza A and B may still be helpful and guide patient management. Secondly, the variations of SARS‐CoV‐2 and influenza viruses were not tested in the current study. Validation of the design across all virus variants was not performed in this study, as it was outside the scope of the TUBITAK‐TEYDEB project. Thirdly, Cross‐reactivity was assessed with Rhinovirus, *S. pyogenes,* and *M. pneumoniae*. Seasonal coronaviruses and parainfluenza viruses were not tested but could be added to future studies. Finally, since this study focuses on assay development rather than producing a kit, performance validation metrics such as sensitivity, specificity, positive predictive value, and negative predictive value were not assessed. These parameters may be evaluated in future clinical validation studies.

## 5. Conclusions

The system offers an efficient approach for multitarget detection, reducing processing time, and simplifying the workflow. Detection of 24 targets in a single well on a 4‐channel device can increase multiplexing capacity. In the study, IC performance was consistent, and specificity was high; clinical accuracy and error reduction can be further evaluated in future studies.

This prototype was first tested on a respiratory syndromic panel containing SARS‐CoV‐2, RSV, and Inf A‐B viruses. The assay has the potential to be expanded and transformed into high‐throughput diagnostic platforms not only in respiratory tract infections but also in many syndromic panels, such as HPV genotyping, sexually transmitted diseases, and gastrointestinal panels.

## Conflicts of Interest

The authors declare no conflicts of interest.

## Author Contributions

Conceptualization: M.S, A.A, and T.A, data curation: M.S, A.A, and T.A; formal analysis: T.A; funding acquisition: M.S; investigation: M.S, A.A, and T.A; methodology: T.A; project administration: M.S; resources: M.S; software: T.A; supervision: M.S; validation: M.S and T.A; visualization: M.S; writing–original draft preparation: A.A and T.A; writing–review and editing: M.S, A.A, and T.A.

## Funding

This study has been supported by the Scientific and Technological Research Council of Turkey (TUBITAK‐TEYDEB) (7220793).

## Data Availability

The data that support the findings of this study are available on request from the corresponding author. The data are not publicly available due to privacy or ethical restrictions.
